# Conjugates of γ-Carbolines and Phenothiazine as new selective inhibitors of butyrylcholinesterase and blockers of NMDA receptors for Alzheimer Disease

**DOI:** 10.1038/srep13164

**Published:** 2015-08-18

**Authors:** Galina F. Makhaeva, Sofya V. Lushchekina, Natalia P. Boltneva, Vladimir B. Sokolov, Vladimir V. Grigoriev, Olga G. Serebryakova, Ekaterina A. Vikhareva, Alexey Yu. Aksinenko, George E. Barreto, Gjumrakch Aliev, Sergey O. Bachurin

**Affiliations:** 1 Institute of Physiologically Active Compounds Russian Academy of Sciences, Chernogolovka, 142432, Russia; 2 Emanuel Institute of Biochemical Physics, Russian Academy of Sciences, Moscow, 119334, Russia; 3 Departamento de Nutrición y Bioquímica, Facultad de Ciencias, Pontificia Universidad Javeriana, Bogotá D.C., Colombia; 4 Instituto de Ciencias Biomédicas, Universidad Autónoma de Chile, Santiago, Chile; 5 GALLY International Biomedical Research Consulting LLC., San Antonio, TX 78229, USA; 6 School of Health Science and Healthcare Administration, University of Atlanta, Johns Creek, GA 30097, USA

## Abstract

Alzheimer disease is a multifactorial pathology and the development of new multitarget neuroprotective drugs is promising and attractive. We synthesized a group of original compounds, which combine in one molecule γ-carboline fragment of dimebon and phenothiazine core of methylene blue (MB) linked by 1-oxo- and 2-hydroxypropylene spacers. Inhibitory activity of the conjugates toward acetylcholinesterase (AChE), butyrylcholinesterase (BChE) and structurally close to them carboxylesterase (CaE), as well their binding to NMDA-receptors were evaluated *in vitro* and *in silico*. These newly synthesized compounds showed significantly higher inhibitory activity toward BChE with IC_50_ values in submicromolar and micromolar range and exhibited selective inhibitory action against BChE over AChE and CaE. Kinetic studies for the 9 most active compounds indicated that majority of them were mixed-type BChE inhibitors. The main specific protein-ligand interaction is π-π stacking of phenothiazine ring with indole group of Trp82. These compounds emerge as promising safe multitarget ligands for the further development of a therapeutic approach against aging-related neurodegenerative disorders such as Alzheimer and/or other pathological conditions.

The development of novel compounds that are able to modify the pathogenesis of neurodegenerative diseases appears to be as a promising approach among different drug discovery strategies in this emerging area^1^,^2^. Taking into account the multifactorial nature of neurodegenerative diseases^3^,^4^, focusing on the design of multitarget drugs that are capable to act simultaneously on different main biotargets, which are involved in the disease pathogenesis, seems to be very attractive and promising^5^,^6^,^7^,^8^. During the past decade, previous studies have indicated that the progression of Alzheimer disease (AD), amyotrophic lateral sclerosis (ALS) and some other neuropathological disorders is closely connected to dysfunctions in cholinergic and glutamatergic neuronal systems^9^,^10^,^11^,^12^. Nowadays, the main scheme for AD treatment is the use of three inhibitors of cholinesterase’s (donepezil, rivastigmine and galantamine) and low-affinity antagonist of NMDA-receptors—memantine^13^. The current standard of AD treatment recommends combination of AChE inhibitors with memantine^13^,^14^,^15^,^16^,^17^,^18^. Recently, it was shown that combining glutamatergic and cholinergic approaches in the symptomatic treatment of AD could be much more efficient^17^ compared to the single treatment option. In this regard, the design of new compounds that can interact with both of these neuromediator systems is more likely to confer better protection against neurodegeneration and therefore compensating the deficit of cholinergic and glutamatergic functions that appeared to be key features of these diseases^19^.

Recent studies showed that Dimebon (latreperdine) and methylene blue (MB) are able to protect neurons in different models of neurodegeneration^20^,^21^,^22^,^23^,^24^. Moreover, significant protective effects were observed in a vitro model of ALS when both compounds are administered simultaneously^25^. In this context, we previously synthesized a group of original compounds that combine γ-carboline fragment of dimebon and phenothiazine core of MB in the same structure^26^,^27^, as a novel approach to the development of multitarget disease-modifying agents (Fig. 1).

In the present work, we assessed the biological action of such compounds on the key targets of cholinergic and glutamatergic systems, in particular, acetylcholinesterase (EC 3.1.1.7, AChE), butyrylcholinesterase (EC 3.1.1.8, BChE) and structurally close to them, carboxylesterase (EC 3.1.1.1, CaE), as well on binding to NMDA-receptors. The background for selection of these biological targets were the results of the previous observations that phenothiazine derivatives including MB can effectively inhibit the enzymes of cholinesterase family^28^,^29^,^30^,^31^ and one of the target of dimebon neuronal action is the NMDA-receptor^32^.

## Results

### Inhibiting activity of conjugates of γ-carboline and phenothiazine against human erythrocyte AChE, equine serum BChE and porcine liver CaE

All γ-carboline-phenothiazine conjugates have been assessed as inhibitors of AChE and BChE, which are important for AD and/or AD-like dementia development and structurally close enzyme—CaE. CaE is responsible for hydrolysis of numerous ester-containing drugs^33^,^34^. Inhibition of CaE by anticholinesterase compounds leads to adverse drug-drug interactions^35^. AChE from human erythrocytes was used along with two enzymes of non-human origin, namely BuChE from horse serum and CaE from porcine liver because of their lower cost, high degree of identity with human enzymes and the exploratory character of this work. The inhibitory potency was described as IC_50_—an inhibitor concentration, which reduces the enzyme activity by half. In our study, dimebon, phenothiazine and methylene blue were used as reference compounds. Bis-4-nitrophenyl phosphate (BNPP), a selective inhibitor of CaE^36^ was used as a positive control in CaE inhibition study.

**Figure 1:**
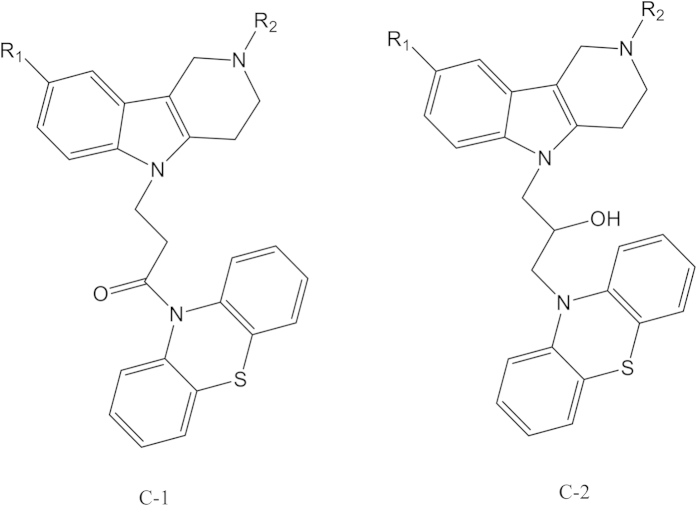
Structures of the studied conjugates of γ-carbolines and phenothiazine. R_1_, R_2_ = Alkyl, F.

The results, which are summarized in Table 1, show that the conjugates with both types of spacers: 1-oxopropylene one (C-1 series) and 2-hydroxypropylene one (C-2 series) (Fig. 1) very weakly inhibited AChE and CaE and had rather high inhibitory activity against BChE. All of the compounds showed submicromolar and micromolar activity and very high selectivity against BChE; they were more active (10–15 times for the most active compounds) and much more selective inhibitors of BChE than that compared to dimebon.

Among the studied conjugates, the maximum inhibitory activity was showed by 1-oxopropylene spacer connected compounds (C-1e) and (C-1f) containing ethyl substituent in carboline cycle (R_2_ = C_2_H_5_): IC_50_ = 0.52 ± 0.01 and 0.58 ± 0.06 μM, respectively. For compounds with 2-hydroxypropylene spacer, the most active was compound (C-2c) with R_1_ = CH_3_O, R_2_ = CH_3_: IC_50_ = 0.39 ± 0.02 μM. Compound (C-1h) with bulky iso-propyl substituent R_1_ was twice less active (IC_50_ = 2.79 ± 0.09 μM) than compound (C-1g) with R_1_ = C_2_H_5_ (IC_50_ = 1.36 ± 0.06 μM). The least active as BChE inhibitors were compounds (C-1a) and (C-2a) with R_1_ = R_2_ = CH_3_.

Comparison of IC_50_ values for the conjugates (C-1f) and (C-2b), which have 1-oxo- and 2-hydroxypropylene spacers and identical substituents R_1_ and R_2_ (R_1_ = F, R_2_ = C_2_H_5_), showed 3.5 times higher inhibitory activity for the compound with 1-oxopropylene spacer. However, as indicated in the Table 1, for the studies with conjugates with different substituents R_1_ and R_2_, anti-BChE activity varies moderately, with maximal activity IC_50_ = 0.39 ± 0.02 μM for compound (C-2c) and minimal one IC_50_ = 2.79 ± 0.09 μM for compound (C-1h).

**Figure 2:**
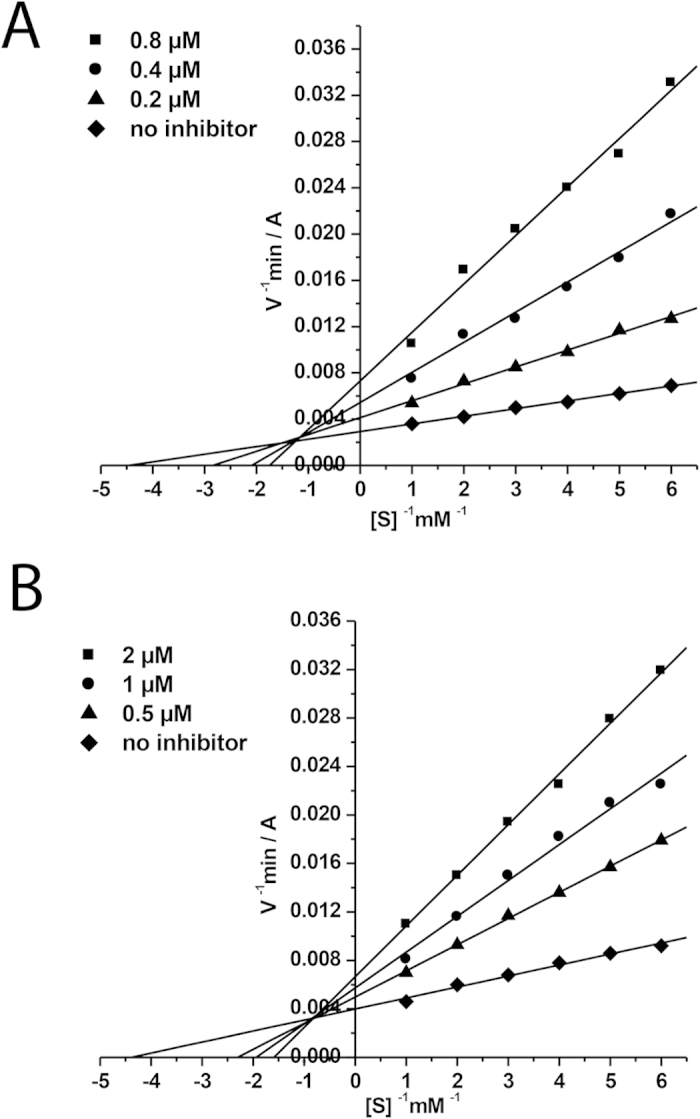
Steady state inhibition of BChE by compounds (C-1f), (**A**) and (C-2b) (**B**). Lineweaver-Burk reciprocal plots of initial velocity and substrate concentrations in the presence of inhibitors (C-1f), (C-2b) (three concentrations) and their absence are presented. The plots A and B show mixed-type inhibition.

The mechanisms of action of the 9 most active compounds (C-1b)—(C-h) and (C-2b)—(C-2c) towards BChE are presented in Table 2. The linear Lineweaver—Burk equation, which is a double reciprocal form of the Michaelis—Menten one, was used to evaluate the selective characteristics and type of inhibition. As an example, the graphical analysis of steady-state inhibition data for compounds (C-1f) and (C-2b) towards BChE is shown in Fig. 2. The compounds have identical substituents R_1_ and R_2_ and different spacers.

Both compounds are mixed-type reversible inhibitors. As shown in Figs 2A,B, binding of compounds (C-1f) and (C-2b) to BChE changed both V_max_ and K_m_ values, a trend that is generally ascribed to mixed-type inhibition. In particular, a decreased V_max_ at increasing inhibitor concentrations and increasing intercepts (higher K_m_) with higher inhibitor concentration were observed. Thus, a structure of the spacer does not affect the mechanism of BChE inhibition by the studied conjugates. The value of inhibition constant for compound (C-1f) was *K*_*i*_ = 0.17 ± 0.02 μM (competitive component) and *αK*_*i*_ = 0.52 ± 0.04 μM (noncompetitive component). For compound (C-2b) *K*_*i*_ = 0.82 ± 0.02 μM (competitive component) and *αK*_*i*_ = 2.98 ± 0.19 μM (noncompetitive component). The values of obtained BChE inhibition constants (*K*_*i*_—component competitive and *αK*_*i*_—non-competitive component) are shown in Table 2. Most of the conjugates of γ-carboline and phenothiazine are mixed-type reversible inhibitors of BChE were seen. Only one compound (C-1h) with bulky *iso*-propyl substituent inhibits BChE by non-competitive mechanism (Table 2).

### Molecular modeling

The nature of high inhibitory activity and selectivity of γ-carboline-phenothiazine conjugates to BChE was assessed by molecular docking of the compounds to BChE active site. In the results below, we show the docking of the most active compounds (C-1f) (R_1_ = F, R_2_ = C_2_H_5_) and (C-2c) (R_1_ = CH_3_O, R_2_ = CH_3_) containing 1-oxo- and 2-hydroxypropylene spacers.

### Quantum mechanical optimization of the ligand structures

**Figure 3:**
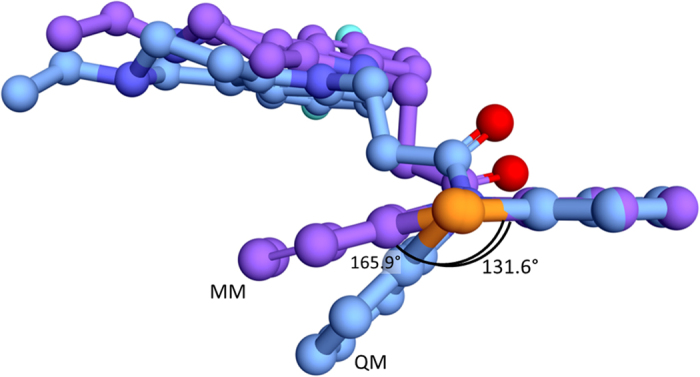
Overlay of structures of phenothiazine fragment of compound (C-1f) after MM (carbon atoms are colored violet) and QM (carbon atoms are colored blue) optimizations.

Geometries of the ligands under consideration were optimized quantum mechanically. Comparing to the initial molecular mechanical (MM) optimization, the geometries changed significantly. Phenothiazine fragment after MM optimization has almost planar shape, while after QM optimization it has geometry of two planes intersect at the angle (Fig. 3). This observation similar to the earlier report was already described in^28^,^37^, and such a shape was called *“butterfly”.*

### Molecular docking

**Figure 4:**
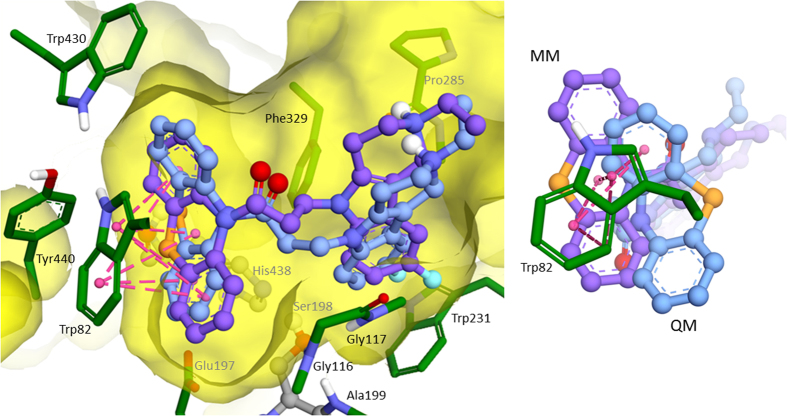
Overlay of the best docked positions of compound (C-1f) into active site of BChE. Carbon atoms of MM optimized structure are shown violet and QM-optimized are colored blue. Views from two different points are shown.

**Figure 5:**
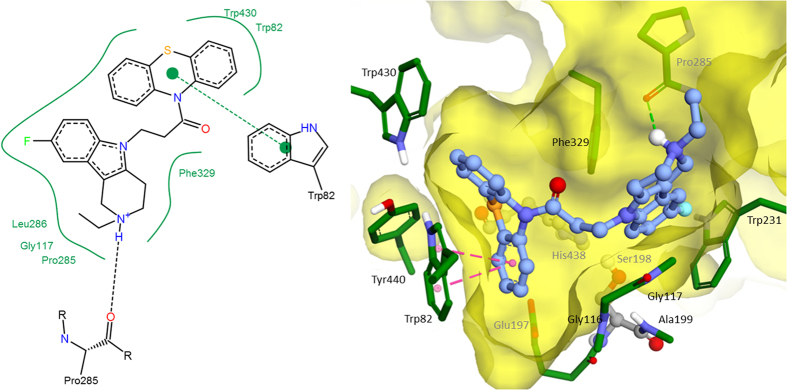
The best docked position of compound (C-1f) inside BChE (2D and 3D images).

**Figure 6:**
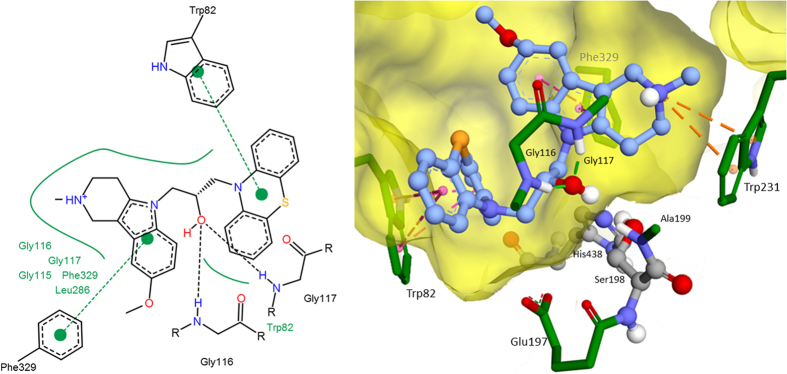
The best docked position of compound (C-2c) inside BChE (2D and 3D images).

Molecular docking was performed for MM and QM optimized structures of compound (C-1f). The calculated binding free energy for MM-optimized geometry −11.89 kcal/mol (the corresponding theoretical *K*_*i*_ = 1.94 nM) was significantly overestimated comparing to the experimental data of *K*_*i*_ = 0.17 ± 0.02 μM, α*K*_*i*_ = 0.52 ± 0.04 μM. For the QM-optimized structure, the estimated binding affinity reduced due to weaker interaction of non-planar phenothiazine fragment with Trp82 comparing to the planar one as a result of decrease of π-systems overlap (Fig. 4). Estimated binding free energy of compound (C-1f) was −8.89 kcal/mol (the theoretical *K*_*i*_ = 0.3 μM) and linearly positively correlated with the experimental data. Consequently, for all compounds QM-optimized structures were used for molecular docking. As a result, estimated binding energies were in −7.5–−9.5 kcal/mol range, which correspond to the experimental inhibitory activity range of 3–0.1 μМ. There were few specific interactions of conjugates of γ-carboline and phenothiazine with active site and gorge of BChE, while it had perfect geometry fitness. The major interaction was π-π stacking between indole ring of Trp82 and phenothiazine fragment, though slightly weakened by non-planarity of the latter (Figs 5, 6). Besides, hydroxyl group of (C-2c) forms hydrogen bonds with the BChE oxyanion center (Fig. 6). Additionally, weak π-π interaction exists between γ-carboline fragment and Phe329. In protonated forms of the ligands, positively charged group might form additional interactions: π-cation in the case of (C-2c) with Trp231 (Fig. 6) and hydrogen bond with Pro285 in the case of (C-1f) (Fig. 5). However, these interactions contribution is not significant, since for the charged form the estimated binding free energy differences was less than 1 kcal/mol.

### Radioligand study of compounds interaction with NMDA-receptor binding sites

We have assessed the interaction of synthesized compounds with two main binding sites of NMDA-receptor, namely with [^3^H] МК-801-binding site and with [^3^H] ifenprodil-binding site (Table 3). For series of compounds with 1-oxopropylene spacer (C-1) the connection of phenothiazine fragment to γ-carboline cycle leads to significant increase (in 10 times) of their affinity towards МК-801 site compared to dimebon. Binding to ifenprodil site increases in some cases. In particular, compounds (C-1e), (C-1f) and (C-1g) containing ethyl substituent in the carboline cycle have the strongest binding characteristics towards both NMDA-receptor sites. When a 2-hydroxypropylene spacer was used (C-2 series), the reduction of the conjugates affinity towards both sites was detected. At the same time, none of compounds showed any selectivity in relation to ifenprodil-binding site typical for MB (Table 3).

## Discussion

In the present study, we provide a throughout evaluation of the inhibitory activity on AChE, BChE and CaE of the γ-carboline-phenothiazine conjugates by kinetics and computational tools as well radioligand assessment of conjugates interaction with two binding sites of NMDA receptor. AChE and BChE are important for AD and/or AD-like dementia development and structurally close enzyme – CaE is responsible for the hydrolysis of numerous ester-containing drugs^33^,^34^. Inhibition of CaE by anticholinesterase compounds leads to adverse drug-drug interactions^35^. In our study, dimebon, phenothiazine and methylene blue were used as reference compounds. The results in Table 1 demonstrate, regardless the type of spacer, the γ-carboline-phenothiazine conjugates had rather high inhibitory activity toward BChE and very low activity against two other studied esterases, AChE and CaE. Thus, the compounds possess a high inhibitory selectivity to BChE. The conjugates were more active (10–15 times for the most active compounds) and much more selective inhibitors of BChE compared to dimebon. That is, substitution of 2-pyridoethyl fragment in a dimebon molecule on phenothiazine group connected to gamma-carboline by 1-oxopropylene or 2-hydroxypropylene spacer changes the esterase profile^38^,^39^ of the dimebon. As for other reference compounds, MB is more specific to AChE than to BChE, while it is well known that its phenothiazine core has been the basis of many selective inhibitors of BChE^28^,^29^,^31^,^37^,^40^. Very low activity of γ-carboline-phenothiazine conjugates against AChE indicates that these compounds will not cause unwanted side effects inherent AChE inhibitors; lacking inhibitor activity against CaE suggests they will not cause adverse drug-drug interactions.

The molecular docking results suggest that interaction between γ-carboline-phenothiazine conjugates and BChE active site and gorge are characterized rather by good geometrical complementarity than those specific interactions. This geometrical fitness seems to be the main reason for high inhibitory activity and selectivity of the compounds under consideration. Since the main specific protein-ligand interaction is π-π stacking between indole group of Trp82 and phenothiazine fragment of conjugates, this explains moderate effect of alterations of structure of γ-carboline fragment and the spacer nature on inhibitory activity of the conjugate compounds.

In healthy brains, acetylcholine is mainly hydrolysed by AChE, while BChE plays a secondary role. However, in AD brains, the activity of AChE decreases while that of BChE gradually rises^41^,^42^. Therefore, BChE appears as an increasingly important therapeutic target to reduce AD cholinergic deficit^41^,^43^,^44^. The remarkable activity and selectivity towards BChE showed by the conjugates (C-1e), (C-1f) and (C-2c) could be of great importance in the development of selective new and more specific anti-AD therapies, since it has been described that selective BChE inhibition increases brain acetylcholine and improves learning in rodents^44^,^45^. Moreover, the proven efficacy of inhibitors affecting both cholinesterases^46^,^47^,^48^ and the clinical failure of AChE-specific inhibition suggest that BuChE inhibition could be important for more effective treatment of AD. Therefore, BuChE-selective inhibitors provide promise for improved clinical benefit^49^.

By our previous observations, the NMDA-receptor is one of the targets of dimebon neuronal action^32^. The radioligand binding study of γ-carboline-phenothiazine conjugates with two main binding sites of non-competitive negative modulators of NMDA-receptor, namely intra-channel blocker МК-801 and allosteric modulator ifenprodil, was performed. It was observed that the ligand properties of conjugates radically differed from dimebon and MB—the basic structures for designed compounds (Table 3). The substitution of 2-pyridoethyl fragment in a dimebon molecule on phenothiazine group connected to γ-carboline by 1-oxopropylene spacer increases (in 10 times) compounds binding to both NMDA-receptor binding sites; whereas using 2-hydroxypropylene spacer impairs binding properties. None of compounds showed any selectivity in relation to ifenprodil-binding site typical for MB. The compounds (C-1e), (C-1f) and (C-1g) containing ethyl substituent in the carboline cycle demonstrated the strongest binding characteristics towards both NMDA-receptor sites. It can be assumed that compounds that compete for them would also act as non-competitive negative modulators of NMDA-receptor. Two of the compounds (C-1e) and (C-1f) also were the best inhibitors of BChE.

## Conclusions

To discover multifunctional agents for treatment of neurodegenerative diseases, a series of original compounds, which combine γ-carboline fragment of dimebon and phenothiazine core of MB in one molecule was studied as inhibitors of AChE, BChE and CaE. It was found that the conjugates had a high inhibitory activity toward BChE with IC_50_ values in submicromolar and micromolar range and exhibited strong inhibitory activities and selectivity against BChE over AChE and CaE. Studies of the compounds binding to МК-801 and ifenprodil-binding sites of NMDA-receptors showed that conjugates with 1-oxopropylene spacer had increased affinity towards both NMDA-receptor binding sites compared to the dimebon. Compounds (C-1e) and (C-1f), which showed the highest affinity to both NMDA-receptor sites, also were significant inhibitors of BChE. It is important to point that these compounds did not inhibit AChE, therefore will not cause unwanted side effects; they also did not inhibit the structurally related enzyme CaE, i.e. and will not cause adverse drug-drug interactions. Finally, these compounds emerge as promising safe multitarget ligands for drugs development against age-related neurodegenerative disorders such as Alzheimer, Parkinson or other related conditions.

## Materials and Methods

### Chemistry

The studied conjugates of γ-carbolines and phenothiazine (Fig. 1) have been synthesized as described previously^26^,^27^.

### Biological assay

#### *In vitro* AChE, BChE and CaE inhibition

Acetylcholinesterase (AChE, EC 3.1.1.7, from human erythrocyte), butyrylcholinesterase (BChE, EC 3.1.1.8, from equine serum), carboxylesterase (CaE, EC 3.1.1.1, from porcine liver), acetylthiocholine iodide (ATCh), butylthiocholine iodide (BTCh), 5,5´-dithiobis-(2-nitrobenzoic acid) (DTNB), 4-nitrophenyl acetate (4-NPA), were purchased from Sigma-Aldrich (Germany).

AChE and BChE activities were measured by the method of Ellman and coworkers as described earlier^50^. The assay solution consisted of 0.1 M K/Na phosphate buffer pH 7.5, 25 °C with the addition of 0.33 mM DTNB, 0.02 unit/mL of AChE or BChE and 1 mM of substrate (ATCh or BTCh, respectively). Assays were carried out with a blank containing all components except ATCh and BTCh in order to account for non-enzymatic reaction.

The activity of CaE was determined spectrophotometrically by the release of 4-nitrophenol at 405 nm^51^. The assay solution consisted of 0.1 M K/Na phosphate buffer pH 8.0, 25 °C with the addition of 1 mM 4-nitrophenyl acetate and 0.02 unit/mL of CaE. Assays were carried out with a blank containing all components except CaE.

The tested compounds were dissolved in DMSO; the incubation mixture contained 2% of the solvent. Eight different concentrations of the test compounds in the range of 10^−11^–10^−4^ M were selected in order to obtain inhibition of AChE and BChE activity comprised between 20% and 80%. The test compounds were added to the assay solution and preincubated at 25 ^°^C with the enzymes for 10 min followed by the addition of substrate. A parallel control was made for the assay solution with no inhibitor. Measurements were performed in a BioRad Benchmark Plus microplate spectrophotometer (France). Each experiment was performed in triplicate. The results were expressed as the mean ± SEM. The reaction rates in the presence and absence of inhibitor were compared, and the percent of residual enzyme activity due to the presence of test compounds was calculated. IC_50_ (the concentration of inhibitor required to decrease the enzyme activity by 50%) values were determined graphically from inhibition curves (log inhibitor concentration vs percent residual enzyme activity) using the Origin 6.1 software.

#### Kinetic analysis of BChE inhibition. Determination of steady-state inhibition constants

To elucidate the inhibition mechanisms for the most active compounds, the BChE residual activity were determined in the presence of 3 increased concentrations of the test compounds and 6 decreasing concentrations of the substrates. The test compounds were preincubated with the enzymes at 25 °C for 10 min, followed by the addition of the substrates. Parallel controls were made for an assay of the rate of hydrolysis of the same concentrations of substrates in the solutions with no inhibitor. The kinetic parameters of substrate hydrolysis were determined. Measurements were performed in a BioRad Benchmark Plus microplate spectrophotometer (France). Each experiment was performed in triplicate. Results were fitted into Lineweaver-Burk double-reciprocal kinetic plots of 1/V versus 1/[S] and values of inhibition constants *K*_*i*_ (competitive component) and *αK*_*i*_ (noncompetitive component) were calculated using the program Origin 6.1.

#### Radioligand study of compounds interaction with NMDA-receptor binding sites

Effect of test compounds on the radioligand binding to NMDA receptors was determined by using a modified method as reported earlier by Zhou L-M and coworkers^52^. Two radioactive ligands were used: [^3^H] MK-801 (dizocilpine) with a specific activity of 210 Ci/mmol binding to all isolated NMDA receptors, and [^3^H] ifenprodil with a specific activity of 79 Ci/mmol binding only to NMDA receptors containing the NR2B subunit^53^,^54^.

A membrane preparation of hippocampus for radioligand analysis was prepared by the techniques described previously^55^. The obtained membrane pellet was resuspended in a work buffer (5 mM HEPES/4.5 mM Tris buffer, pH 7.6) in a ratio of 1:5, and stored in liquid nitrogen. The reaction mixture (the final volume of 0.5 ml) contained 200 μl of the working buffer, 50 μl of 50 nM radioligand solution and 250 μl of the membrane suspension. Nonspecific binding was determined in the presence of 50 μl of 1 M of unlabeled ligand.

For binding study, the reaction mixture was incubated at room temperature for 2 hours. After incubation, the samples were filtered through the glass-fiber filters GF/B (Whatman), washed with the work buffer, dried and transferred to scintillation vials to which 5 ml of scintillation fluid was added containing 4g diphenyl oxazole (PPO), 0.2g diphenyloxazoil benzene (POPOP) and 1 liter of toluene. Radioactivity was determined in the scintillation counter TriCarb2800 TR (PerkinElmer, Packard, USA) with counting efficiency of about 65%.

Investigation of the effect of the tested compounds on the binding of [^3^H] MK-801 and [^3^H] ifenprodil to rat hippocampal membranes was carried out by adding to the incubation medium 50 μl of the test compounds in the concentration range of 10^−8^–10^−3^ M. By the results of inhibition, IC_50_ values were calculated for the tested compounds using GraphPadPrism 4 Demo. In the cases where inhibition by the test compound in the concentration of 100 mM did not exceed 50%, the value of IC_50_ was not determined (n/d).

### Molecular modelling

To determine protonation state of piperidine nitrogen atom of γ-carboline fragment of the compounds, Marvin 6.3.0 (ChemAxon, http://www.chemaxon.com) was used to estimate p*K*_a_ values. Since they were found to be close to 7.4, forms, protonated and neutral were used for molecular docking.

Geometries of the ligands were quantum-mechanically (QM) optimized in Gamess-US package^56^ using DFT method B3LYP and basis 6-31G*. Partial atomic charges were taken from QM results according to Mulliken scheme^57^. The PDB^58^ structure of human BChE 1P0I^59^ was used. Previously the importance of saturation of BuChE gorge with water molecules was demonstrated^60^. Protein structure was prepared, saturated with water molecules and optimized using QM/MM method as reported previously^60^,^61^.

Molecular docking with a Lamarckian Genetic Algorithm^62^ was performed with Autodock 4.2.6 software^63^. Grid box for docking included the whole BChE active site and the gorge with dimensions 15 Å × 20.25 Å × 18 Å with grid spacing 0.375 Å. The main of selected Lamarckian Genetic Algorithm parameters were 256 runs, 25 × 10^6^ evaluations, 27 × 10^4^ generations and population size 300. For the best docked positions, additional 256 runs of local search were performed. Docking positions with the lowest binding energies were used for analysis. Structural images were prepared with Accelrys Discovery Studio Visualiser 4.0 (http://www.accelrys.com), 2D images of protein-ligand are prepared with PoseView software (http://poseview.zbh.uni-hamburg.de/).

## Additional Information

**How to cite this article**: Makhaeva, G. F. *et al.* Conjugates of γ-Carbolines and Phenothiazine as new selective inhibitors of butyrylcholinesterase and blockers of NMDA receptors for Alzheimer Disease. *Sci. Rep.* **5**, 13164; doi: 10.1038/srep13164 (2015).

## Figures and Tables

**Table 1:** Inhibitory activity (IC_50_) of conjugates of γ-carbolines and phenothiazine (Fig. 1) towards AChE, BChE and CaE.

Compounds			IC_50_ (μM) ± SEM		
No	R_1_	R_2_	AChE	BChE	CaE
C-1a	CH_3_	CH_3_	>200	62.6 ± 4.3	>200
C-1b	CH_3_	С_2_H_5_	>200	2.04 ± 0.55	>200
C-1c	F	CH_3_	>200	1.79 ± 0.28	>200
C-1d	H	CH_3_	>200	1.07 ± 0.12	>200
C-1e	H	С_2_H_5_	>200	0.52 ± 0.01	>200
C-1f	F	С_2_H_5_	>200	0.58 ± 0.06	>200
C-1g	С_2_H_5_	CH_3_	>100	1.36 ± 0.06	>200
C-1h	i-С_3_H_7_	CH_3_	>100	2.79 ± 0.09	120 ± 13
C-2a	CH_3_	CH_3_	n.a.	11.7 ± 0.4	>200
C-2b	F	С_2_H_5_	n.a.	2.01 ± 0.04	>200
C-2c	CH_3_O	CH_3_	n.a.	0.39 ± 0.02	>200
dimebon			36.3 ± 5.8	5.76 ± 0.51	n.a.
phenothiazine			n.a.	137 ± 31	n.a.
МB			1.21 ± 0.09	11.1 ± 0.1	>200
BNPP			n.a.	n.a.	1.80 ± 0.11

**Table 2:** Inhibition constants of the active conjugates of γ-carbolines and phenothiazine (Fig. 1) towards BChE^a^.

Compounds			*K*_*i*_ (μM)	α*K*_*i*_ (μM)
No	R_1_	R_2_		
C-1b	CH_3_	С_2_H_5_	0.43 ± 0.05	1.46 ± 0.40
C-1c	F	CH_3_	0.48 ± 0.06	1.27 ± 0.41
C-1d	H	CH_3_	0.37 ± 0.01	1.64 ± 0.20
C-1e	H	С_2_H_5_	0.26 ± 0.02	0.65 ± 0.07
C-1f	F	С_2_H_5_	0.17 ± 0.02	0.52 ± 0.04
C-1g	С_2_H_5_	CH_3_	0.46 ± 0.02	0.99 ± 0.04
C-1h	i-С_3_H_7_	CH_3_		1.94 ± 0.01
C-2b	F	С_2_H_5_	0.82 ± 0.02	2.98 ± 0.19
C-2c	CH_3_O	CH_3_	0.25 ± 0.04	0.89 ± 0.18
MB			0.35 ± 0.01	0.64 ± 0.02

^a^Values for *K*_*i*_ (competitive inhibition constant) and α*K*_*i*_ (non-competitive inhibition constant) were determined from analysis of slopes of 1/V versus 1/S at various inhibitor concentrations. Values (means ± SEM) are from at least three experiments.

**Table 3:** The binding of γ-carboline-phenothiazine conjugates (Fig. 1) to МК-801 and ifenprodil binding sites of NMDA receptor.

Compounds			Binding characteristics of compounds			
No	R_1_	R_2_	% of [^3^H]МК-801 blockadeat 100 μM ofcompound	[^3^H]МК-801, IC_50_, μM	% of [^3^H]ifenprodilblockade at100 μM ofcompound	[^3^H]ifenprodil,(IC_50_, μM)
C-1a	CH_3_	CH_3_	80.4 ± 6.6	13.5 ± 3.6	42.6 ± 7.2	88.4 ± 8.3
C-1b	CH_3_	С_2_H_5_	95.7 ± 1.0	8.5 ± 0.8	48.1 ± 5.9	74.4 ± 4.0
C-1c	F	CH_3_	78.7 ± 1.4	17.7 ± 2.6	50.2 ± 5.2	55.1 ± 5.8
C-1d	H	CH_3_	74.9 ± 1.1	18.5 ± 0.9	60.1 ± 1.4	23.4 ± 0.7
C-1e	H	С_2_H_5_	76.1 ± 1.2	14.6 ± 1.9	69.4 ± 3.3	13.4 ± 2.6
C-1f	F	С_2_H_5_	82.4 ± 7.3	15.8 ± 1.8	78.2 ± 6.8	8.8 ± 1.8
C-1g	С_2_H_5_	CH_3_	89.4 ± 4.6	13.2 ± 2.2	64.6 ± 5.2	15.4 ± 3.9
C-1h	i-С_3_H_7_	CH_3_	80.3 ± 6.9	17.8 ± 2.0	88.7 ± 5.6	85.8 ± 7.2
C-2a	CH_3_	CH_3_	87.1 ± 7.7	84.8 ± 9.2	69.7 ± 8.3	57.2 ± 6.7
C-2b	F	С_2_H_5_	24.1 ± 1.9	106.3 ± 9.2	25.7 ± 4.6	81.2 ± 6.7
C-2c	CH_3_O	CH_3_	19.1 ± 2.2	113.4 ± 11.1	47.4 ± 6.6	115.4 ± 9.3
dimebon			27.8 ± 3.9	91.5 ± 7.7	34.1 ± 4.9	82.4 ± 4.1
МB			2.0 ± 4.0	n/d	70.4 ± 10.1	9.3 ± 4.5
